# Application of Deep Eutectic Solvents for the Extraction of Carnosic Acid and Carnosol from Sage (*Salvia officinalis* L.) with Response Surface Methodology Optimization

**DOI:** 10.3390/plants10010080

**Published:** 2021-01-02

**Authors:** Martina Jakovljević, Stela Jokić, Maja Molnar, Igor Jerković

**Affiliations:** 1 Faculty of Food Technology Osijek, Josip Juraj Strossmayer University of Osijek, Franje Kuhača 18, 31000 Osijek, Croatia; mjakovljevic@ptfos.hr (M.J.); sjokic@ptfos.hr (S.J.); mmolnar@ptfos.hr (M.M.); 2 Faculty of Chemistry and Technology, University of Split, Ruđera Boškovića 35, 21000 Split, Croatia

**Keywords:** sage, optimization, stirring and heating extraction, ultrasound-assisted extraction, mechanochemical extraction

## Abstract

*Salvia officinalis* L. is a good source of antioxidant compounds such as phenolic diterpenes carnosic acid and carnosol. From 17 deep eutectic solvents (DESs) used, choline chloride: lactic acid (1:2 molar ratio) was found to be the most suitable for the extraction of targeted compounds. The influence of H_2_O content, extraction time, and temperature (for stirring and heating and for ultrasound-assisted extraction (UAE)), H_2_O content, extraction time, and vibration speed for mechanochemical extraction on the content of targeted compounds were investigated. Carnosic acid content obtained by the extraction assisted by stirring and heating was from 2.55 ± 0.04 to 14.43 ± 0.28 µg mg^−1^, for UAE it was from 1.62 ± 0.29 to 14.00 ± 0.02 µg mg^−1^, and for mechanochemical extraction the yield was from 1.80 ± 0.02 to 8.26 ± 0.45 µg mg^−1^. Determined carnosol content was in the range 0.81 ± 0.01 to 4.83 ± 0.09 µg mg^−1^ for the extraction with stirring and for UAE it was from 0.56 ± 0.02 to 4.18 ± 0.05 µg mg^−1^, and for mechanochemical extraction the yield was from 0.57 ± 0.11 to 2.01 ± 0.16 µg mg^−1^. Optimal extraction conditions determined by response surface methodology (RSM) were in accordance with experimentally demonstrated values. In comparison with previously published or own results using conventional solvents or supercritical CO_2_, used DES provided more efficient extraction of both targeted compounds.

## 1. Introduction

The growth of the pharmaceutical industry and increased need for bioactive components has led to the increased development of new extraction and isolation methods [1]. The most important differences between these methods are better efficiency and shorter extraction time for modern techniques compared to the conventional ones. Furthermore, conventional solvents are very often flammable and toxic with their manufacture depending on fossil resources [2]. However, there are also certain issues associated with the modern techniques, such as poor selectivity and solubility of targeted components in the solvents used, such as H_2_O, ethanol, or CO_2_, as well as recovery of bioactive components and their chemical changes during the extraction period due to the reactions such as ionization, hydrolysis, and oxidation [3,4]. Over the past few years, deep eutectic solvents (DESs), first proposed by Abbott et al. [5,6], have been developed as analogues of ionic liquids (ILs), although they differ from them in the starting material and the method of preparation. DESs are mixtures of hydrogen bond acceptor (HBA) and hydrogen bond donor (HBD) with a lower melting point relative to the starting components. Their green character is also attributed to their low price, easy preparation, biodegradability, and low toxicity [7]. In addition to these properties, different studies reported that DES could dissolve several components better than organic solvents, due to dissolving lignocellulose which causes damage of plant cell wall and strengthens the mass transfer process [8]. DESs can be prepared from different combinations of the starting compounds, thus being tunable solvents with different functionality and solubility for various compounds. Therefore, a suitable combination of the starting solvents and their molar ratio can increase the solubility and the extraction efficiency of DESs for desired compounds [9]. However, their shortcomings should also be taken into account with an emphasis on viscosity and low vapor pressure which makes it difficult to isolate and purify desired components. In addition, high viscosity complicates industrial application due to the high energy consumption needed to ensure their liquid state [10].

One of the most commonly used HBA is choline chloride (ChCl), since it is an inexpensive, biodegradable, and non-toxic quaternary ammonium salt. ChCl can form DES with different nontoxic components as carboxylic acids, sugars, sugar alcohols, or amines which act as HBDs [5,6]. In the last few years, deep eutectic solvents are increasingly used for the extraction of phenolic compounds including phenolic acids, flavonoids, stilbenes, anthocyanins, and furanocoumarins [1,2,11,12,13]. In the research performed by Bi et al. [11] DESs were used to extract flavonoids such as myricetin and amentoflavone from *Chamaecyparis obtusa* (Siebold and Zucc.) Endl. leaves by alcohol-based DESs. Wang et al. [13] extracted polyphenols and furanocoumarins from fig (*Ficus carica* L.) with tailor-made DESs and showed that DESs were effective for the extraction of these components. Anthocyanins in the flower petals of *Catharanthus roseus* L. were extracted with natural deep eutectic solvents (NADESs) such as lactic acid—glucose and propane-1,2-diol—choline chloride, which provided higher stability for anthocyanins [12]. The extraction with DES can be improved by combination with ultrasound (UAE), microwaves and heating as well as by mechanochemical extraction (MCE). Therefore, Bosiljkov et al. [14] successfully extracted anthocyanins with UAE combined with DESs. Wang et al. [4] developed fast, efficient, and ecofriendly MCE for tanshinones as well as bioactive compounds from tea leaves [15]. Due to the desirability of DESs in the extraction of phenolic components, we decided to extract phenolic diterpenes, carnosic acid, and carnosol from sage (*Salvia officinalis* L.) applying DESs. These compounds have been suggested to account for over 90% of the sage antioxidant properties [16]. In addition to antioxidant activity, carnosic acid, and carnosol showed proapoptotic [17], antiproliferative [18], anti-angiogenic [19] and antitumor activity. So far, there are no available data on DESs extraction and optimization of the parameters for carnosic acid and carnosol from sage. There are few reports dealing with DESs extraction of sage by Bakirtzi et al. [2] and Georgantzi et al. [20] who investigated the influence of different DESs on the extraction of polyphenols from medicinal plants including sage. In paper by Bakirtzi et al. [2] lactic acid-based natural deep eutectic solvents in combination with ultrasound were used for the extraction of total polyphenols and flavonoids from sage, while Georgantzi et al. [20] investigated combination of lactic acid-based DES with cyclodextrin for UAE of total polyphenols and flavonoids.

Taking into account all the above mentioned, the objectives of this study were focused on (1) investigation on finding appropriate choline chloride based deep eutectic solvent for the extraction of carnosic acid and carnosol as well as (2) suitable extraction techniques (stirring with heating, UAE or MCE). Afterwards, the influence of various DES extraction parameters (H_2_O content, time, and temperature for stirring and heating and UAE; H_2_O content, extraction time and vibration speed for mechanochemical extraction) on the (3) content of carnosic acid and carnosol in sage extract analyzed by HPLC was investigated. In addition, (4) the optimal extraction conditions by RSM for desired antioxidant components (carnosic acid and carnosol) were determined.

## 2. Results and Discussion

### 2.1. Influence of DESs on the Obtained Amount of Carnosic Acid and Carnosol in the Extracts

**Figure A1:**
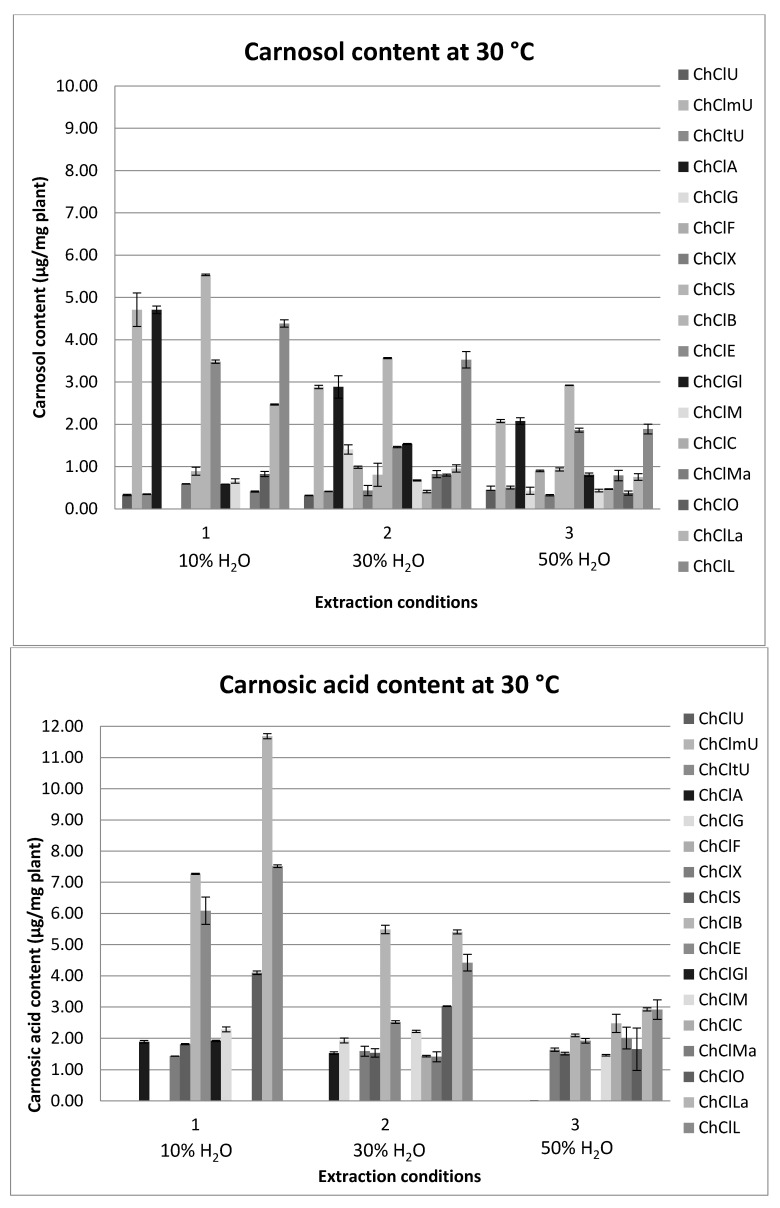
Carnosol and carnosic acid content obtained by stirring and heating with DESs depending on the temperature and H_2_O content (*n* = 3). The columns represent determined amount of carnosol and carnosic acid in the samples, and the color of the column indicates DES used according to the abbreviations recorded in the legend to the right and Table 4.

Due to the different effects of viscosity, surface tension, polarity, and HBD interaction it is hard to estimate the suitability of a DES for the extraction of targeted compounds. Therefore, in order to select the best DES for the extraction of carnosic acid and carnosol from sage, the extraction was performed with different solvents and different H_2_O addition at 30 °C (Figure A1). According to Dai et al. [12] and Bosiljkov et al. [14], H_2_O addition in organic acid-based DESs causes decrease of the solvent polarity since these solvents are more polar than H_2_O. Therefore, for targeted components it would be more suitable to lower H_2_O addition (which is consistent with the results obtained). As can be seen from Figure A1, the solvents substantially differ in their ability to extract carnosic acid and carnosol. In addition to the influence of HBD, the amount of H_2_O added also plays an important role for the extraction efficiency. For certain solvents, like choline chloride:malonic acid (1:1 molar ratio), the amount of carnosic acid was increased with increased H_2_O content that may be due to the viscosity lowering effect. For DES choline chloride:citric acid (1:1 molar ratio), the highest amount of carnosic acid and carnosol was obtained with 30% H_2_O addition that may be the consequence of a high viscosity of the solvent with 10% H_2_O addition. H_2_O amount was changed to reduce the viscosity which causes a slow mass transfer, thus affecting the extraction process. The viscosity of DESs can be reduced by the addition of a certain amount of H_2_O as well as by increasing the temperature [12]. Several solvents such as choline chloride:glucose (1:1 molar ratio) were too viscous even with the addition of H_2_O at 50% (*v*/*v*). Although the addition of H_2_O can decrease the viscosity, an excessive concentration of H_2_O can decrease the interactions between the components of DES as well the interactions between DES and desired components [11]. This is the reason why the focus was on H_2_O addition in the range of 10–50% (*v*/*v*).

Carnosol can be extracted with all DESs applied, but choline chloride based DESs with butane-1,4-diol (1:2 molar ratio) (ChClB) and ethane-1,4-diol (1:2 molar ratio) (ChClE) were the most effective. The result of extraction with choline chloride:glucose (1:1 molar ratio) with 10% H_2_O were not shown because of the high viscosity preventing further analysis. The same limitation was observed with choline chloride:fructose (1:1 molar ratio) (ChClF) and choline chloride:citric acid (1:1 molar ratio) (ChClC) at 10% of H_2_O and at 30 °C. The higher amount of carnosol was observed at lower H_2_O content (Figure A1). With choline chloride:urea (1:2) (ChClU) as the solvent, the highest amount of carnosol was extracted at 30 °C. According to the literature, higher content of carnosol is usually present in the extracts obtained at higher temperatures since it is one of the degradation products of carnosic acid [20].

On the other hand, not all applied solvents could extract carnosic acid. Basic solvents, such as ChCl:U, choline chloride:*N*-methlyurea (1:3 molar ratio) (ChClmU), and choline chloride:thiourea (1:2 molar ratio) (ChCltU), were not efficient for carnosic acid extraction. The highest amount of carnosic acid was extracted using acidic DESs, with emphasis on choline chloride:lactic acid (1:2 molar ratio) (ChClLa). Such solvents are significantly acidic (pH < 3) compared to the solvents where HBDs were sugars or alcohols (pH > 6) [7,12]. Additionally, the polarity of DESs should also be considered as an important criterion for the evaluation and selection of the solvents to achieve maximum extraction efficiency. Carnosic acid and carnosol are polar constituents, soluble in polar solvents (which is in agreement with the obtained results), such as DESs with organic acids as HBDs, which are more polar than the DESs with sugars as HBDs [12]. The highest amount of carnosic acid was obtained with the lowest amount of added H_2_O (10%) in most solvents. Okamura et al. [21] have investigated the effect of temperature on the degradation of carnosic acid in acetone solution and reported that the increase of temperature affected the degradation of carnosic acid. In case of the extraction with DESs such as ChClLa and choline chloride:levulinic acid (1:2 molar ratio) (ChClL) the maximum amount of carnosic acid was extracted at 30 °C.

Since carnosol can be extracted with all DESs applied and due to the highest amount of extracted carnosic acid in the case of choline chloride:lactic acid (1:2 molar ratio) and the lactic acid properties as natural component, this solvent was selected for further optimization of the extraction with three extraction methods (stirring and heating, UAE, and MCE).

### 2.2. Comparison of the Used Extraction Methods

After selection of the appropriate solvent, the extractions performed by stirring and heating and UAE, applying the same temperature and H_2_O content as well as MCE were compared. Both, stirring and heating and UAE increase mass transfer and speed up diffusion of the compounds. In the case of ultrasound, acoustic cavitation phenomenon leads to the disruption of cell walls and consequently improves the yield of extraction compared to maceration [22]. However, MCE can decrease the processing time and solvent consumption and reduce noise and radiation compared to UAE and to stirring and heating extraction.

Table 1 shows that slightly higher amounts of carnosol and carnosic acid were extracted by stirring and heating, compared to UAE. Such results can be explained by the positive influence of stirring on the mass transfer in such viscous solvent. For MCE, the utilization of the glass beads led to much better mixing of the plant material and solvent, thus extracting significant amounts of carnosic acid and carnosol in a shorter time compared to the other two extractions (Table 2).

For the constant H_2_O content in all extraction methods, the extracted amounts of carnosic acid and carnosol obtained by different extractions were compared. The extracted amounts of selected compounds (8.26 and 7.92 µg mg^−1^ for carnosic acid and 1.87 and 2.02 µg mg^−1^ for carnosol) obtained by MCE at Run 12 and 17 can be compared to Run 6 and 9 obtained with stirring and heating extraction and UAE. The difference between the parameters of these extractions was the extraction time, so at 10% H_2_O for MCE, 2 min were enough to obtain similar amounts of targeted compounds as for 30 min of stirring and heating extraction and UAE. In the case of 30% H_2_O by MCE, 3 min were sufficient to obtain the amount of extracted components similar to the amount extracted for 90 min by stirring and heating extraction and UAE. It is important to note that MCE was carried out at room temperature (24–28 °C). In fact, by using mill, we wanted to show how much time was needed for the extraction at room temperature, and prolonging the extraction time would result in warming of the samples without the possibility of heating control.

### 2.3. Influence of Various DES Extraction Parameters on the Content of Carnosol and Carnosic Acid

The effect of H_2_O addition, temperature or vibration speed and extraction time on carnosol and carnosic acid was investigated for three extraction techniques using DES choline chloride:lactic acid (1:2 molar ratio). In these experiments, the content of carnosic acid in sage extract obtained by stirring and heating was 2.55–14.43 µg mg^−1^, depending on the applied extraction parameters. The lowest content of carnosic acid was obtained at 50% (*v*/*v*) H_2_O added at 30 °C and 60 min, while the highest content was obtained at 10% (*v*/*v*) H_2_O added at 50 °C and 90 min (Table 1). The content of carnosic acid obtained by UAE varied, depending on the parameters used, in the range 1.62–13.99 µg mg^−1^. The lowest content of carnosic acid was obtained at 30% (*v*/*v*) H_2_O added, 70 °C, and 30 min and the highest yield at 10% (*v*/*v*) H_2_O added, 70 °C and 60 min (Table 1). The content of carnosic acid obtained by MCE varied depending on the parameters used in the range 1.80–8.26 µg mg^−1^. The lowest content of carnosic acid was at 50% (*v*/*v*) H_2_O added, vibration speed of 1 m/s and 2 min and the highest yield at 10% (*v*/*v*) H_2_O added, 5 m/s and 2 min (Table 2). The content of carnosol obtained by mixing and heating was 0.81–4.83 µg mg^−1^ depending on the applied extraction parameters. The lowest content of carnosol was obtained at 50% (*v*/*v*) H_2_O addition, 50 °C and 30 min, while the highest yield was obtained at 10% (*v*/*v*) of H_2_O, 50 °C and 90 min. The content of carnosol, depending on the parameters used in UAE, was 0.56–4.18 µg mg^−1^ with the lowest content at 30% (*v*/*v*) H_2_O addition, 30 °C and 30 min and the highest yield at 10% H_2_O addition, 70 °C and 60 min (Table 1). The content of carnosol obtained by MCE was 0.57–2.02 µg mg^−1^ depending on the applied extraction parameters (Table 2). The lowest content of carnosol was obtained at 50% of H_2_O (*v*/*v*), vibration speed of 1 m/s and time of 1 min, while the highest content was obtained at 30% of H_2_O (*v*/*v*), 5 m/s and 3 min.

**Figure 1:**
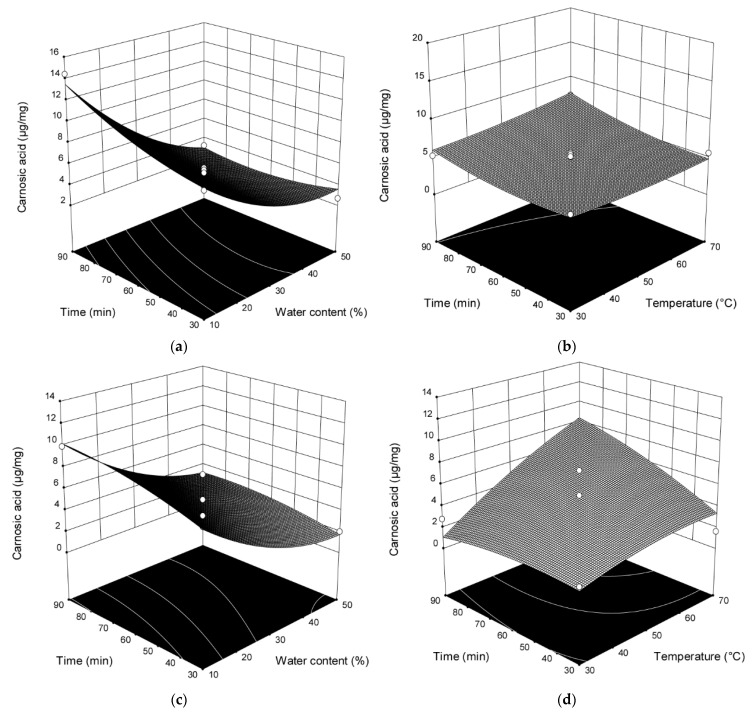

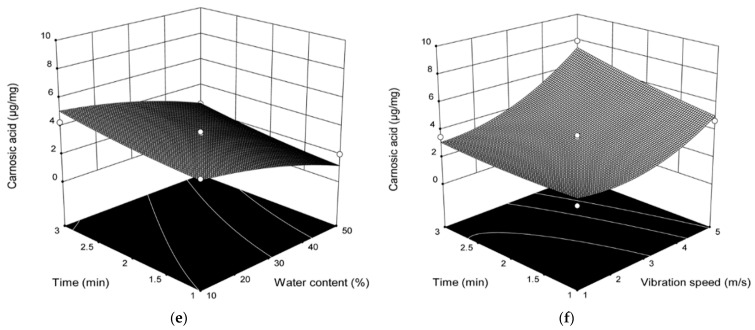
Three-dimensional plots for obtained content of carnosic acid as a function of the extraction time, temperature, and H_2_O content for the extraction with mixing and heating (**a**,**b**), UAE (**c**,**d**) and for the extraction time, vibrational speed, and H_2_O content for MCE (**e**,**f**).

The addition of H_2_O and extraction time (Figure 1 and Table A1) showed statistically significant influence on the content of carnosic acid (*p* < 0.0001; *p* = 0.0202) in the extracts obtained by stirring and mixing. The content of carnosic acid increased with prolonged extraction time and decreased with the increase of H_2_O amount.

The interactions between amount of H_2_O added and extraction time (*p* = 0.0259) also showed a significant influence on the content of carnosic acid. In the extracts obtained by UAE, H_2_O addition and temperature showed statistically significant influence on the content of carnosic acid (*p* = 0.0025; *p* = 0.0144). For this extraction technique, interactions between the amount of added H_2_O and temperature (*p* = 0.0433) also showed a significant influence in terms of content of carnosic acid. The content of carnosic acid increased with increased extraction temperature and decreased with the increase of H_2_O amount. In the extracts obtained by MCE, H_2_O addition, time, and vibration speed (*p* = 0.0006; *p* = 0.0266; *p* = 0.0002) as well as the interactions between H_2_O addition and vibration speed (*p* = 0.0221) showed statistically significant influence on the content of carnosic acid. The content of carnosic acid increased with prolonged extraction time and vibration speed and decreased with the increase of H_2_O amount.

**Figure 2:**
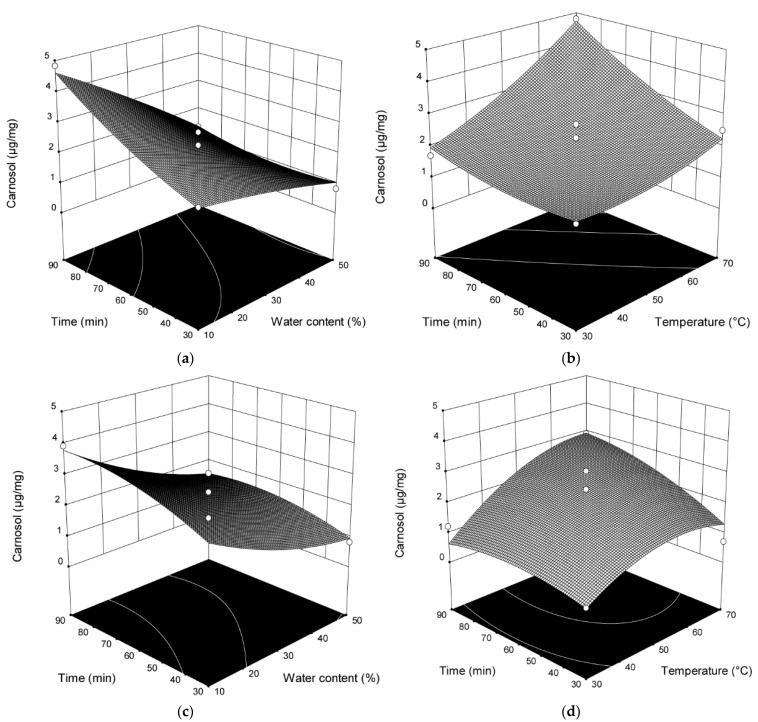
Three-dimensional plots for obtained content of carnosol as a function of the extraction time, temperature, and H_2_O content in the extraction with mixing and heating (**a**,**b**) and MCE (**c**,**d**).

As can be seen from Figure 2 and Table A2, H_2_O addition, extraction time, and temperature showed statistically significant influence on the content of carnosol (*p* < 0.0001; *p* = 0.0008; *p* = 0.0003) in the extracts obtained by stirring and mixing. The content of carnosol is increased with increased time and temperature of the extraction and with decreased H_2_O amount.

Interactions between amount of H_2_O added and the extraction time and between the amount of H_2_O added and temperature (*p* = 0.0184; *p* = 0.0234) also showed a significant influence for the content of carnosol. In the extracts obtained by MCE, H_2_O addition, time, and vibration speed (*p* = 0.0055; *p* = 0.0187; *p* = 0.0012) showed statistically significant influence on the content of carnosol. The content of carnosol increased with prolonged extraction time and vibration speed and decreased with the increase of H_2_O amount. Since model according to RSM is not significant for the extraction of carnosol with ultrasound (*p* = 0.0708), the results obtained for that extraction are not discussed. To optimize the extraction conditions of two different phenolic diterpenes 17 runs determined by BBD with three variables (percentage of H_2_O added, time and temperature or vibration speed) at three levels were used to fit a second-order response surface. The amount of carnosic acid and carnosol were observed as the response (Table A1 and Table A2).

The data describing the optimal conditions for the extraction of carnosic acid and carnosol from sage using DESs are not available in the literature, but there are few papers investigating the optimal conditions with other solvents. In paper by Fatma Ebru et al. [23] it was shown that 70% of ethanol was the most efficient solvent since it extracted 3.45 mg carnosol + carnosic acid per g of the extract. According to the optimization carried out, they showed that the amount of these bioactive components was in the function of extraction time. In addition, they also demonstrated that carnosol and carnosic acid degraded easily at higher temperatures over a longer period of time. Therefore, they have shown that the optimum conditions were temperatures of 40–50 °C, the extraction time 3–6 h, solvent-to-sage ratio 6:1 (*v*/*w*) and 70–80 wt.% ethanol for maceration. Similar results were also showed in paper by Durling et al. [24]. According to the optimization carried out, the amount of targeted components depended on several parameters such as particle size, temperature, time, and a solvent-to-sage ratio. The highest concentration of targeted components was obtained with the particle size 1 mm, 40 °C, the extraction time of 3 h, the solvent-to-sage ratio of 6:1 (*v/w*) and 55–75 wt.% ethanol. Under these conditions, the extract containing 10.6% carnosic compounds was obtained.

The optimization process of extraction is important for determining the most favorable conditions for achieving maximum yields of desired components in the extracts. Based on BBD, estimated coefficients of second order response models for carnosol and carnosic acid in sage extracts are given in Table A1 and Table A2. *R*^2^ for carnosic acid was 0.9630 and for carnosol was 0.9607 in the extracts obtained by stirring and heating, and for UAE *R*^2^ for carnosic acid was 0.8660. In the case of MCE, *R*^2^ for carnosic acid was 0.9442 and for carnosol *R*^2^ was 0.9032. According to ANOVA, statistically significant models for carnosic acid (Table A3) and carnosol in the extraction by stirring and heating (Table A4) (*p* ≤ 0.05) were obtained. Additionally, the obtained models showed non-significant lack of fit (*p* = 0.2042–0.4491), except in the case of MCE for carnosic acid (*p* = 0.0008).

According to RSM, optimum conditions are expressed as those at which it is possible to achieve the maximum amount of carnosic acid and carnosol. They are slightly different depending on the extraction technique used, so for the extraction with heating and mixing they were 10% H_2_O addition, 90 min and 70 °C, while for UAE they were 11.05% of H_2_O addition, 82.36 min and 69.84 °C. Under these optimal conditions, the content of carnosic acid and carnosol was calculated as 14.20 µg mg^−1^ and 6.47 µg mg^−1^ in case of stirring and heating and 14.72 µg mg^−1^ of carnosic acid for ultrasound extraction. The desirability for these optimizations was 0.990 and 1.0, respectively. The experimental results for the amount of carnosic acid and carnosol obtained at optimum conditions were 13.73 ± 0.26 and 6.15 ± 0.33 µg mg^−1^ for the extraction with stirring and heating, while for UAE this amount was 14.24 ± 0.21 µg mg^−1^. Optimum conditions for MCE were 11.13% H_2_O addition, time of extraction 2.90 min, and vibration speed 4.98 m s^−1^. Under these optimal conditions, the content of carnosic acid and carnosol is calculated as 8.95 µg mg^−1^ and 2.02 µg mg^−1^ with the desirability 1.0 which was confirmed experimentally (8.90 ± 0.10; 2.03 ± 0.04 µg mg^−1^).

### 2.4. Comparison with Other Extraction Methods

According to the literature, the most common solid–liquid extraction of sage has been performed with methanol. Due to the toxic effect of methanol, it is preferable to use ethanol which can be classified as bio-solvent and is much safer for the use [25,26]. In the paper by Abreu et al. [27] the content of carnosic acid and carnosol in methanolic extract of sage was 14.6 mg g^−1^ of dry weight and 0.4 mg g^−1^, respectively. This is similar to our results for Run 2 (mixing and heating) and Run 3 (UAE), but with a significantly higher amount of carnosol in our case. Sage extraction with 80% methanol over 24 h at room temperature led to the extraction of carnosic acid only with the content of 273.8 mg 100 g^−1^ of the plant dry weight [28], much lower than our results. In other case, the extraction with 50% methanol during 60 min in ultrasound bath has brought carnosic acid content of 2.1 g kg^−1^ extract and carnosol content of 4.1 to 15.1 mg g^−1^ of plant dry weight [29].

According to Table 3, which shows our results obtained by the stirring with heating extraction of the same sage material with common solvents, it is observed that the most effective solvent is absolute ethanol, while H_2_O is the least effective solvent for the extraction of carnosic acid and carnosol. The preparation of aqueous solutions of ethanol in the range of 30–70% (*v*/*v*) shows that the increase in the volume of ethanol (*v*/*v*) increased the amount of extracted components. In this case, methanol as the extraction solvent shows lower extraction efficiency compared to ethanol. In addition, the influence of extraction parameters such as extraction time and temperature can be observed in Table 3. However, when ethanol is used as the extraction solvent and with the most efficient extraction conditions applied (50 ° C and 90 min), lower amount of carnosic acid and carnosol was obtained compared to the selected DES (choline chloride:lactic acid 1:2).

Considering the adverse properties of organic solvents and in order to overcome their disadvantages, such as low selectivity for antioxidant compounds [30], safe or green solvents and processes have been used. Supercritical fluid extraction (SFE) has been used in the plant material extraction due to its ability to provide clean extracts without residual solvent [31]. In addition, SFE can be performed at low temperatures in short time, which is suitable for carnosic acid oxidation prevention during the extraction, also supported by the absence of air and light during the extraction process thus reducing its degradation [32]. In our previous work [33] we used the same herbal material for carnosic acid and carnosol extraction using SFE with CO_2_ (SC-CO_2_). Comparing the results, the highest amount of extracted carnosic acid using SC-CO_2_ was 855.8 mg 100 g^−1^ of the plant material (30 MPa, 50 °C, 1 kg h^−1^ CO_2_), while the extraction yield using DESs was 1443.22 mg 100 g^−1^ and 1399.22 mg 100 g^−1^, depending on the extraction technique employed. In the case of carnosol, the highest amount was extracted under the same conditions of SC-CO_2_ (446.35 mg 100 g^−1^), and similar results were achieved using DESs (483.34 and 418.39 mg 100 g^−1^ of plant, depending on the extraction technique employed). However, certified reference material was not used and therefore minor changes in the composition of the plant material are possible with respect to the same sample used in our previously published data. In the paper published by Babovic et al. [34] the content of carnosic acid obtained by SC-CO_2_ was 13.76 g per 100 g of the extract and carnosol content was 6.97 g per 100 g of the extract similar to our results (11.63 g carnosic acid per 100 g and 8.55 g carnosol per 100 g) [32]. Despite the fact that SC-CO_2_ extraction conditions may reduce carnosic acid degradation, we still notice that more carnosic acid was extracted and preserved by DESs extraction even at higher extraction temperatures. On the other hand, preparing DESs is simple and inexpensive, i.e., the price is comparable to the cost of the conventional solvents. Moreover, this is sustainable process theoretically without generated waste [10] which makes this extraction process suitable for the extraction of bioactive components including carnosic acid and carnosol.

## 3. Materials and Methods

### 3.1. Chemicals

The standard compounds carnosic acid (≥95.0%) and carnosol (99.2%) (Sigma Chemical Co., St. Louis, MO, USA) were used for the chemical analyses. All solvents were of analytical grade and purchased from J.T. Baker (Avantor, Phillipsburg, NJ, USA).

### 3.2. Plant Material

Dried sage leaves (*Salvia officinalis* L.) were used for experiments. Prior to the extraction, the dried leaves were grounded and sieved using a vertical vibratory sieve shaker (LabortechnikGmbh, Ilmenau, Germany) as described in paper by Jokić et al. [35].

### 3.3. Preparation of DES

The choline chloride based DESs were prepared as described in our paper [36]. In this study, seventeen different choline chloride based DESs were prepared using inexpensive components as shown in Table 4.

### 3.4. Extraction of Carnosic Acid and Carnosol with DESs

Grounded *Salvia officinalis* L. dried leaves (50 mg) were mixed with 1 mL of the selected solvent, a pure DES or a mixture of DES and ultrapure H_2_O (Millipore Simplicity 185, Darmstadt, Germany). Prepared samples were stirred at 1500 rpm in aluminum block (Stuart SHB) on a magnetic stirrer or ultrasound treated in temperature-controlled ultrasonic bath at specified temperature for the certain time (Table 1). The temperature-controlled ultrasonic bath (Elma P70 H, Singen, Germany) was set with frequency at 37 Hz and power at 50 W at the same temperature over the same time as in case of mixing in aluminum block (Table 1). Prepared samples (50 mg of plant + 1 g of glass beads with 1 mL of solvent) were also extracted on the BeadRuptor 12 ball mill (Omni International, Kennesaw, GA, USA) according to parameters in Table 2 at room temperature (24–28 °C). After the extraction, the mixture was centrifuged for 15 min and then decanted. The supernatant liquid was then diluted with methanol, filtered through a PTFE 0.45 μm filter, and subjected to HPLC analysis.

### 3.5. Extraction of Carnosic Acid and Carnosol with Conventional Solvents

Grounded *Salvia officinalis* L. dried leaves (50 mg) were mixed with 1 mL of selected solvent (Millipore Simplicity 185, Darmstadt, Germany). Prepared samples were stirred at 1500 rpm in aluminum block (Stuart SHB) on a magnetic stirrer at specified temperature for the certain time (Table 3).

### 3.6. Chemical Characterization of the Extracts

HPLC analyses of carnosic acid and carnosol was performed on an Agilent 1260 Infinity II (Agilent, Santa Clara, California, USA) with chromatographic separation obtained on a ZORBAX Eclipse Plus C18 (Agilent, Santa Clara, CA, USA) column (100 × 4.6 mm, 5 µm).

**Figure A2:**
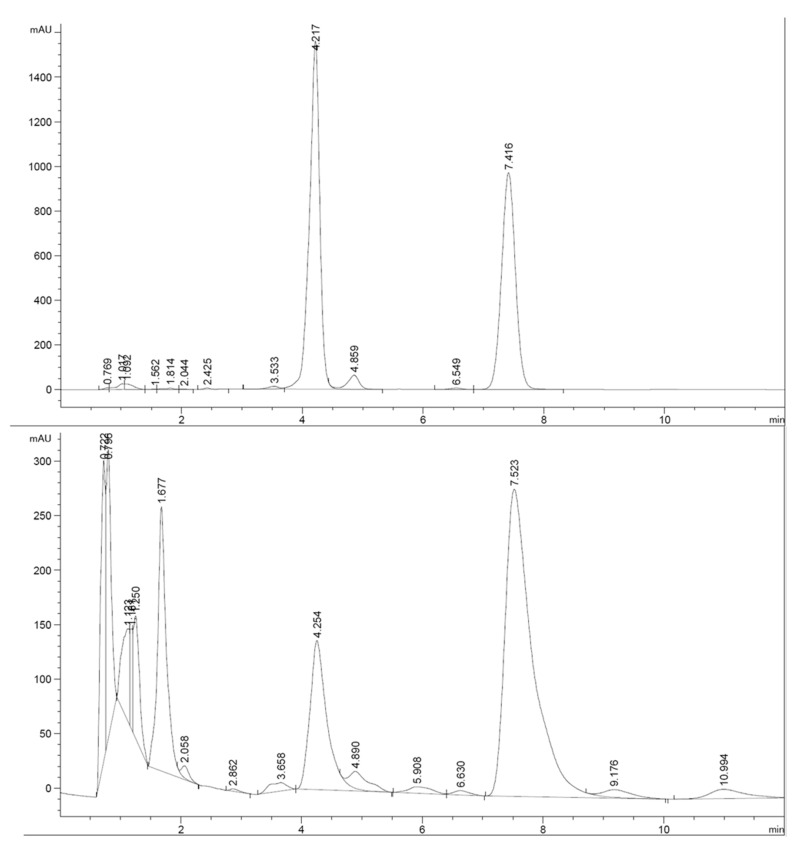
Obtained chromatogram of carnosol and carnosic acid standards (picture **above**) and chromatogram of real sample (picture **below**).

The separation of analyzed compounds was made with method described in our previous paper [31], but since analysis was performed on different device, linearity of the calibration curve, LOQ and LOD was confirmed. Standard stock solutions for carnosic acid and carnosol were prepared in a methanol and calibration was obtained at eight concentrations (concentration range 10.0, 20.0, 30.0, 50.0, 75.0, 100.0, 150.0, and 200.0 mg L^−1^). Due to *R*^2^ = 0.99789 for carnosic acid and *R*^2^ = 0.99968 for carnosol, calibration curve was confirmed. Limit of detection were 0.795 mg L^−1^ and 0.971 mg L^−1^ for carnosic acid and carnosol, respectively. Limit of quantification were 2.648 mg L^−1^ and 7.416 mg L^−1^ for carnosic acid and carnosol, respectively. Retention time for carnosic acid was 7.416 min, while for carnosol was 4.217 min. The chromatograms of the standard and real sample are shown in the Appendix A (Figure A2). For the validation of the HPLC method for the determination of carnosic acid and carnosol, in addition to linearity, retention time comparison and absorption spectrum comparison with standards, repeatability of measurements and solution preparation as well as accuracy were performed, which is also shown in the Appendix A (Table A5).

### 3.7. Statistical Experimental Design

BBD explained in detail by Bas and Boyaci [37] was used for determination of optimal DES (stirring and heating), UAE-DES and MCE-DES extraction conditions in terms of getting higher amount of carnosic acid and carnosol in the *S. officinalis* extracts. Independent variables in design were H_2_O content (X_1_), time (X_2_) and temperature (X_3_) and vibration speed (X_3_) and tested levels were reported in Table 5. Design-Expert^®^ Commercial Software (ver. 9, Stat-Ease Inc., Minneapolis, MN, USA) was used for data analysis. The analysis of variance (ANOVA) was also used to evaluate the quality of the fitted model, and the test of statistical difference was based on the total error criteria with a confidence level of 95.0%.

## 4. Conclusions

In present study, determination of suitable deep eutectic solvent and optimization of the extraction of carnosol and carnosic acid from sage were performed. Among 17 different solvents, choline chloride:lactic acid (1:2 molar ratio) was selected for the extraction by heating and mixing, as well as for ultrasound and mechanochemical extraction. The content of carnosic acid and carnosol was slightly higher in the extracts obtained by stirring and heating and mechanochemical extraction. The influence of H_2_O content, extraction time and temperature (for stirring and heating and for ultrasound-assisted extraction (UAE)), H_2_O content, extraction time and vibration speed for mechanochemical extraction on the content of targeted compounds were investigated. Optimal extraction conditions determined by response surface methodology (RSM) were in accordance with experimentally demonstrated values.

Compared to SC-CO_2_ extraction, we observed that more carnosic acid is extracted using DESs, with emphasis on ChClLa, while the amount of carnosol detected in the extract obtained by ChClLa is similar to that obtained by SC-CO_2_. In addition, the comparison with the solvents such as ethanol, H_2_O, aqueous solutions of ethanol (30–70% (*v*/*v*)) and methanol under the same extraction conditions, showed that choline chloride:lactic acid (1:2 molar ratio) was more efficient for the extraction of carnosic acid and carnosol compared to used conventional solvents.

Given the amounts of carnosic acid achieved at high temperatures in DES in further research it would be useful to examine the stability of the component over the certain period of time.

## Figures and Tables

**Table 1:** Experimental matrix and values (µg mg^−1^ of the plant material) of observed response for the extraction with choline chloride:lactic acid (1:2 molar ratio) obtained by stirring with heating and by ultrasound-assisted extraction (UAE). The results are expressed as mean value ± standard deviation (*n* = 3).

Run	H_2_O (%)	Time (min)	Temperature (°C)	DES-MIXING		DES-UAE	
				Carnosic Acid (µg mg^−1^)	Carnosol (µg mg^−1^)	Carnosic Acid (µg mg^−1^)	Carnosol (µg mg^−1^)
**1**	30	60	50	3.86 ± 0.82	1.99 ± 0.05	7.37 ± 0.34	3.06 ± 0.21
**2**	10	90	50	14.43 ± 0.28	4.83 ± 0.09	9.94 ± 0.22	3.92 ± 0.09
**3**	10	60	70	10.16 ± 0.05	3.90 ± 0.37	14.00 ± 0.02	4.18 ± 0.05
**4**	30	60	50	4.53 ± 0.49	2.27 ± 0.09	4.18 ± 0.21	2.22 ± 0.14
**5**	30	60	50	5.16 ± 0.45	2.70 ± 0.17	5.04 ± 0.05	2.46 ± 0.29
**6**	10	30	50	8.71 ± 0.26	2.10 ± 0.06	8.48 ± 0.06	3.34 ± 0.10
**7**	30	30	70	5.64 ± 0.11	2.51 ± 0.22	1.62 ± 0.29	0.70 ± 0.02
**8**	50	90	50	3.16 ± 0.14	1.34 ± 0.21	2.09 ± 0.37	0.84 ± 0.29
**9**	30	90	70	7.41 ± 0.06	4.79 ± 0.38	8.06 ± 0.13	2.93 ± 0.02
**10**	10	60	30	10.68 ± 0.66	2.35 ± 0.03	2.66 ± 0.41	0.83 ± 0.38
**11**	50	60	70	3.87 ± 0.19	1.85 ± 0.15	3.69 ± 0.24	1.85 ± 0.11
**12**	50	30	50	2.68 ± 0.26	0.81 ± 0.01	2.00 ± 0.07	0.82 ± 0.13
**13**	30	60	50	5.65 ± 0.36	1.99 ± 0.09	4.37 ± 0.09	2.25 ± 0.17
**14**	50	60	30	2.55 ± 0.04	0.89 ± 0.03	1.94 ± 0.21	0.92 ± 0.09
**15**	30	60	50	5.44 ± 0.21	1.79 ± 0.11	2.46 ± 0.05	1.27 ± 0.16
**16**	30	90	30	5.27 ± 0.18	1.70 ± 0.08	2.80 ± 0.25	1.22 ± 0.12
**17**	30	30	30	5.37 ± 0.01	1.50 ± 0.19	2.18 ± 0.23	0.56 ± 0.02

**Table 2:** Experimental matrix and values (µg mg^−1^ of the plant material) of observed response for the extraction with choline chloride:lactic acid (1:2 molar ratio) obtained by mechanochemical extraction (MCE). The results are expressed as mean value ± standard deviation (*n* = 3).

Run	H_2_O (%)	Time (min)	Vibration Speed (m s^−1^)	Carnosic Acid (µg mg^−1^)	Carnosol (µg mg^−1^)
**1**	30	3	1	3.50 ± 0.35	1.25 ± 0.02
**2**	30	2	3	3.38 ± 0.20	1.23 ± 0.06
**3**	50	3	3	2.51 ± 0.34	0.86 ± 0.02
**4**	50	1	3	2.01 ± 0.16	0.71 ± 0.04
**5**	50	2	5	2.98 ± 0.02	1.03 ± 0.13
**6**	10	3	3	4.29 ± 0.25	1.16 ± 0.04
**7**	50	2	1	1.80 ± 0.02	0.57 ± 0.11
**8**	10	2	1	3.37 ± 0.17	1.02 ± 0.20
**9**	10	1	3	4.06 ± 0.26	1.04 ± 0.37
**10**	30	1	5	4.69 ± 0.61	1.27 ± 0.11
**11**	30	2	3	3.31 ± 0.42	1.01 ± 0.09
**12**	10	2	5	8.26 ± 0.45	1.87 ± 0.33
**13**	30	2	3	3.63 ± 0.18	1.14 ± 0.01
**14**	30	2	3	3.45 ± 0.28	1.09 ± 0.12
**15**	30	1	1	2.45 ± 0.07	0.79 ± 21
**16**	30	2	3	3.41 ± 0.24	0.98 ± 0.03
**17**	30	3	5	7.92 ± 0.29	2.02 ± 0.14

**Table 3:** The values (µg mg^−1^ of the plant material) of carnosic acid and carnosol for the extraction obtained by stirring with heating. The results are expressed as mean value ± standard deviation (*n* = 3).

Solvent	Time (min)	Temperature (°C)	Carnosic Acid (µg mg^−1^)	Carnosol (µg mg^−1^)
30% ethanol (*v*/*v*)	30	30	1.11 ± 0.03	2.63 ± 0.21
	60		1.33 ± 0.55	2.26 ± 0.02
	90		1.45 ± 0.53	2.18 ± 0.20
	30	50	2.32 ± 0.07	2.04 ± 0.05
	60		2.64 ± 0.04	1.99 ± 0.04
	90		2.26 ± 0.30	1.51 ± 0.00
	30	70	2.82 ± 0.03	2.92 ± 0.16
	60		2.13 ± 0.14	2.92 ± 0.311
	90		0.93 ± 0.17	2.27 ± 0.38
50% ethanol (*v*/*v*)	30	30	5.91 ± 0.19	4.65 ± 0.22
	60		3.07 ± 0.39	9.31 ± 0.29
	90		3.02 ± 0.15	9.73 ± 0.86
	30	50	7.17 ± 0.05	8.39 ± 0.47
	60		3.15 ± 0.02	9.06 ± 0.11
	90		2.11 ± 0.15	11.25 ± 0.35
	30	70	7.63 ± 0.44	6.73 ± 0.38
	60		4.43 ± 0.20	8.79 ± 0.79
	90		1.91 ± 0.00	11.23 ± 0.13
70% ethanol (*v*/*v*)	30	30	8.28 ± 0.53	3.04 ± 0.01
	60		7.40 ± 0.05	4.82 ± 0.29
	90		7.63 ± 0.0	5.93 ± 0.22
	30	50	7.73 ± 0.22	6.17 ± 0.59
	60		8.54 ± 0.28	7.21 ± 0.44
	90		8.71 ± 0.28	5.43 ± 0.51
	30	70	8.73 ± 0.14	4.37 ± 0.07
	60		7.53 ± 0.06	6.55 ± 0.22
	90		6.85 ± 0.32	6.89 ± 0.26
ethanol	30	30	11.21 ± 0.51	2.72 ± 0.27
	60		11.13 ± 0.13	3.57 ± 0.25
	90		12.77 ± 0.22	2.83 ± 0.06
	30	50	11.74 ± 0.09	3.57 ± 0.47
	60		12.80 ± 0.19	3.14 ± 0.06
	90		13.64 ± 0.10	3.47 ± 0.01
	30	70	13.36 ± 0.37	4.46 ± 0.11
	60		12.27 ± 0.11	3.29 ± 0.21
	90		11.27 ± 0.05	3.11 ± 0.09
methanol	30	30	8.71 ± 0.87	2.88 ± 0.48
	60		9.26 ± 0.06	4.44 ± 0.19
	90		10.50 ± 0.58	4.69 ± 0.58
	30	50	9.68 ± 0.25	5.03 ± 0.08
	60		11.85 ± 0.05	5.24 ± 0.07
	90		10.72 ± 0.30	4.85 ± 0.40
	30	70	9.69 ± 0.58	3.67 ± 0.24
	60		10.11 ± 0.43	4.41 ± 0.10
	90		10.41 ± 0.12	4.80 ± 0.03
H_2_O	30	30	0.00	0.00
	60		0.00	0.24 ± 0.00
	90		0.00	0.24 ± 0.00
	30		0.00	0.29 ± 0.01
	60		0.00	0.27 ± 0.00
	90		0.75 ± 0.03	0.24 ± 0.00
	30		0.00	0.26 ± 0.03
	60		0.00	0.25 ± 0.01
	90		0.74 ± 0.00	0.42 ± 0.02

**Table 4:** Preparation of deep eutectic solvents (DESs).

Name	Combination	Molar Ratio
ChClU	Choline chloride:urea	1:2
ChClmU	Choline chloride:N-methylurea	1:3
ChCltU	Choline chloride:thiourea	1:2
ChClG	Choline chloride:glucose	1:1
ChClF	Choline chloride:fructose	1:1
ChClX	Choline chloride:xylitol	1:1
ChClS	Choline chloride:sorbitol	1:1
ChClB	Choline chloride:butane-1,4-diol	1:2
ChClE	Choline chloride:ethane-1,2-diol	1:2
ChClGl	Choline chloride:glycerol	1:2
ChClA	Choline chloride:acetamide	1:2
ChClM	Choline chloride:malic acid	1:1
ChClC	Choline chloride:citric acid	1:1
ChClMa	Choline chloride:malonic acid	1:1
ChClO	Choline chloride:oxalic acid	1:1
ChClLa	Choline chloride:lactic acid	1:2
ChClL	Choline chloride:levulinic acid	1:1

**Table 5:** Coded and real levels of independent variables for the designed experiment.

Type of Extraction	Independent Variable	Symbol	Level		
			Low (−1)	Middle (0)	High (+1)
Stirring and heating	H_2_O (%)	X_1_	10	30	50
	Time (min)	X_2_	30	60	90
	Temperature (°C)	X_3_	30	50	70
Ultrasound assisted extraction	H_2_O (%)	X_1_	10	30	50
	Time (min)	X_2_	30	60	90
	Temperature (°C)	X_3_	30	50	70
Mechanochemical extraction	H_2_O (%)	X_1_	10	30	50
	Time (min)	X_2_	1	2	3
	Vibration speed (ms^−1^)	X_3_	1	3	5

## Data Availability

The data presented in this study are available from the authors.

## Appendix A

**Table A1:** Regression coefficient of polynomial function of all response surfaces for carnosic acid.

Terms	Coefficients	Standard Error	*F*-Value	*p*-Value
***Carnosic acid (stirring)***				
Intercept	4.93	0.42		
X_1_	−3.97	0.33	145.19	0.0001
X_2_	0.98	0.33	8.95	0.0202
X_3_	0.40	0.33	1.49	0.2619
X_1_^2^	1.60	0.45	12.51	0.0095
X_2_^2^	0.71	0.45	2.46	0.1608
X_3_^2^	0.28	0.45	0.39	0.5530
X_1_X_2_	−1.31	0.47	7.94	0.0259
X_1_X_3_	0.46	0.47	0.98	0.3562
X_2_X_3_	0.47	0.47	1.00	0.3495
*R*^2^*=* 0.9630				
***Carnosic acid (UAE)***				
Intercept	4.76	0.87		
X_1_	−3.17	0.69	21.24	0.0025
X_2_	1.08	0.69	2.45	0.1617
X_3_	2.22	0.69	10.45	0.0144
X_1_^2^	1.39	0.95	2.14	0.1871
X_2_^2^	−0.52	0.95	0.30	0.6019
X_3_^2^	−0.57	0.95	0.37	0.5645
X_1_X_2_	−0.34	0.97	0.12	0.7349
X_1_X_3_	−2.39	0.97	6.06	0.0433
X_2_X_3_	1.46	0.97	2.24	0.1780
*R*^2^*=* 0.8660				
***Carnosic acid (MCE)***				
Intercept	3.44	0.28		
X_1_	−1.34	0.22	35.66	0.006
X_2_	0.63	0.22	7.82	0.0266
X_3_	1.59	0.22	50.46	0.0002
X_1_^2^	−0.38	0.31	1.50	0.2608
X_2_^2^	0.16	0.31	0.27	0.6209
X_3_^2^	1.05	0.31	11.49	0.0116
X_1_X_2_	0.066	0.32	0.043	0.8409
X_1_X_3_	−0.93	0.32	8.56	0.0221
X_2_X_3_	0.54	0.32	2.95	0.1294
*R*^2^ = 0.9442				

**Table A2:** Regression coefficient of polynomial function of all response surfaces for carnosol.

Terms	Coefficients	Standard Error	*F*-Value	*p*-Value
***Carnosol (stirring)***				
Intercept	2.15	0.16		
X_1_	−1.07	0.13	70.76	0.0001
X_2_	0.72	0.13	31.74	0.0008
X_3_	0.86	0.13	45.54	0.0003
X_1_^2^	−0.093	0.18	0.28	0.6146
X_2_^2^	0.22	0.18	1.52	0.2580
X_3_^2^	0.26	0.18	2.26	0.1768
X_1_X_2_	−0.55	0.18	9.34	0.0184
X_1_X_3_	−0.22	0.18	1.48	0.2630
X_2_X_3_	0.52	0.18	8.34	0.0234
*R*^2^*=* 0.9607				
***Carnosol (MCE)***				
Intercept	1.09	0.077		
X_1_	−0.24	0.061	15.65	0.0055
X_2_	0.19	0.061	9.28	0.0187
X_3_	0.32	0.061	27.85	0.0012
X_1_^2^	−0.18	0.084	4.61	0.0690
X_2_^2^	0.029	0.084	0.12	0.7436
X_3_^2^	0.21	0.084	6.33	0.0401
X_1_X_2_	6.381 × 10^−3^	0.086	5.511 × 10^−3^	0.9429
X_1_X_3_	−0.096	0.086	1.26	0.2988
X_2_X_3_	0.074	0.086	0.74	0.4189
*R*^2^*=* 0.9032				

**Table A3:** Analysis of variance (ANOVA) of the modelled responses for carnosic acid.

Source	Sum of Squares	Degree of Freedom	Mean Square	*F*-Value	*p*-Value *
*Carnosic acid (mixing)*					
*The recovery*					
Model	157.64	9	17.52	20.22	0.0003
Residual	6.06	7	0.87		
Lack of fit	3.92	3	1.31	2.44	0.2042
Pure error	2.14	4	0.54		
Total	163.70	16			
*Carnosic acid (UAE)*					
*The recovery*					
Model	171.12	9	19.01	5.03	0.0224
Residual	26.48	7	3.78		
Lack of fit	13.99	3	4.66	1.49	0.3444
Pure error	12.49	4	3.12		
Total	197.60	16			
*Carnosic acid (MCE)*					
*The recovery*					
Model	47.49	9	5.28	13.17	0.0013
Residual	2.81	7	0.40		
Lack of fit	2.75	3	0.92	64.44	0.008
Pure error	0.057	4	0.014		
Total	50.30	16			

******p* < 0.01 highly significant; 0.01 ≤ *p* < 0.05 significant; *p* ≥ 0.05 not significant.

**Table A4:** Analysis of variance (ANOVA) of the modelled responses for carnosol.

Source	Sum of Squares	Degree of Freedom	Mean Square	*F*-Value	*p*-Value *
*Carnosol (mixing)*					
*The recovery*					
Model	22.28	9	2.48	19.04	0.0004
Residual	0.91	7	0.13		
Lack of fit	0.41	3	0.14	1.09	0.4491
Pure error	0.50	4	0.13		
Total	23.19	16			
*Carnosol (UAE)*					
*The recovery*					
Model	18.29	9	2.03	3.18	0.0708
Residual	4.48	7	0.64		
Lack of fit	2.81	3	0.94	2.24	0.2256
Pure error	1.67	4	0.42		
Total	22.77	16			
*Carnosol (MCE)*					
*The recovery*					
Model	1.93	9	0.21	7.26	0.0080
Residual	0.21	7	0.030		
Lack of fit	0.17	3	0.055		
Pure error	0.042	4	0.010		
Total	2.14	16			

******p* < 0.01 highly significant; 0.01 ≤ *p* < 0.05 significant; *p* ≥ 0.05 not significant.

**Table A5:** HPLC method validation parameters.

	Carnosic Acid (µg mg^−1^)	Mean Value (µg mg^−1^)	SD	RSD (%)	Carnosol (µg mg^−1^)	Mean Value (µg mg^−1^)	SD	RSD (%)	
Repeatability of measurements	100.406	100.39	0.43	0.43%	99.999				
	100.042				100.004				
	100.913				100.100				
	100.842				99.947				
	100.305				99.977				
	99.831				99.987	100.00	0.05	0.05%	
Repeatability of solution preparation	104.65	107.46	2.40	2.24%	102.231				
	108.45				101.996				
	109.22				103.260				
	109.09				102.161				
	109.24				101.677				
	104.13				101.996	102.22	0.54	0.53%	
		Added amount of carnosic acid (µg mg^−1^)	Expected value of carnosic acid (µg mg^−1^)	Obtained value of carnosic acid (µg mg^−1^)	Recovery (%)	Added amount of carnosol (µg mg^−1^)	Expected value of carnosol (µg mg^−1^)	Obtained value of carnosol (µg mg^−1^)	Recovery (%)
**Accuracy**	Sample			10.36				6.50	
	Sample + std 1 (1:1)	22.570	16.461	16.426	99%	21.329	13.915	13.213	95%
	Sample + std 2 (1:1)	100.406	55.385	55.797	99%	99.947	53.223	52.999	99%
	Sample + std 3 (1:1)	212.859	111.608	107.049	96%	209.221	107.860	106.995	99%
